# Exploring the Role of Simulation Training in Improving Surgical Skills Among Residents: A Narrative Review

**DOI:** 10.7759/cureus.44654

**Published:** 2023-09-04

**Authors:** Swizel Ann Cardoso, Jenisha Suyambu, Javed Iqbal, Diana Carolina Cortes Jaimes, Aamir Amin, Jarin Tasnim Sikto, Melissa Valderrama, Simranjit Singh Aulakh, Venkata Ramana, Behram Shaukat, Tirath Patel

**Affiliations:** 1 Major Trauma Services, University Hospital Birmingham National Health Service (NHS) Foundation Trust, Birmingham, GBR; 2 Medical Education, Jonalta School of Medicine, University of Perpetual Help System Dalta, Laspinas City, PHL; 3 Neurosurgery, Mayo Hospital, Lahore, PAK; 4 Epidemiology, Universidad Autónoma de Bucaramanga, Bucaramanga, COL; 5 Medicine, Pontificia Universidad Javeriana, Bogotá, COL; 6 Cardiothoracic Surgery, Guy's and St Thomas National Health Service (NHS) Foundation Trust, London, GBR; 7 College of Medicine, Jahurul Islam Medical College and Hospital, Kishoreganj, BGD; 8 Medicine, Pontifica Universidad Javeriana, Cali, COL; 9 Internal Medicine, Ternopil National Medical University, Ternopil, UKR; 10 Orthopedics, All India Institute of Medical Sciences, Mangalagiri, IND; 11 Surgery, Kabir Medical College, Peshawar, PAK; 12 College of Medicine, American University of Antigua, St. John, ATG

**Keywords:** virtual reality simulation, virtual simulations, simulation, surgical, surgery resident performance, skill training, simulation validity, laparoscpic surgery, simulation based continuing education (sbce), skills and simulation training

## Abstract

The role of simulation in medical education is crucial to the development of surgeons' skills. Surgical simulation can be used to improve surgical skills in a secure and risk-free environment. Animal models, simulated patients, virtual reality, and mannequins are some types of surgical simulation. As a result, feedback encourages students to reflect on their strengths and weaknesses, enabling them to focus on improvement. Healthcare simulation is a strong educational instrument, and the main goal of this is to give the students an opportunity to do a practical application of what they have learned through theory. Before taking it to the patients, they will already have certain tools they have previously acquired during the practice. This makes it easier for students to identify the knowledge gaps that they must fill to improve patient outcomes. Moreover, simulation brings a wonderful opportunity for students to acquire skills, gain confidence, and experience success before working with real patients, especially when their clinical exposure is limited.

The use of simulation to teach technical skills to surgical trainees has become more prevalent. The cost of setting up a simulation lab ranges from $100,000 to $300,000. There are several ways to evaluate the effectiveness of simulation-based surgical training. Repetitive surgical simulation training can improve speed and fluidity in general surgical skills in comparison to conventional training. Few previous studies compared learners who received structured simulation training to a group of trainees who did not receive any simulation training in single-center randomized control research. Significantly faster and less time-consuming skill proficiency was noticeable in simulated trainees. Despite being anxious in the operating room for the first time, simulated trainees completed the surgery on time, demonstrating the effectiveness of surgical simulation training.

Traditional surgical training involves senior-surgeon supervision in the operating room. In simulation-based training, the trainees have full control over clinical scenarios and settings; however, guidance and assessment are also crucial. Simulators allow users to practice tasks under conditions resembling real-life scenarios. Simulators can be compared with traditional surgical training methods for different reasons. For example, intraoperative bleeding may occasionally show up not only visibly on the screen but also by shaking the trocars erratically. Without haptics, training on virtual simulators can cause one's pulling and pushing forces, which are frequently greater than what the tissue needs, to be distorted. A good method of simulation training is using virtual reality simulators with haptics and simulated patients. The availability of these facilities is limited, though, and a typical session might include an exercise involving stacking sugar cubes and box trainers. The degree of expertise or competency is one area that needs clarification as medical education transitions to a competency-based paradigm.

The article aims to provide an overview of simulation, methods of simulation training, and the key role and importance of surgical simulation in improving skills in surgical residents.

## Introduction and background

For a surgeon to overcome learning curves and become proficient in several different types of surgeries, they must endure hours of surgical training. The Halsted apprenticeship model of "see one, do one, teach one" has historically served as the foundation for surgical training, but this paradigm has become superfluous due to the evolution of surgical training over the past few decades [1]. 

One of the most crucial aspects of medical education is simulation, as it enables students to acquire the necessary skills in a setting that doesn't compromise the safety of patients while also maximizing their learning potential [2]. This educational strategy offers chances for frequent, secure medical training in a simulated setting. Animal models, simulated patients, virtual reality, and static or interactive mannequins are effectively utilized in simulation programs [3]. Surgeons may instruct and provide feedback in real time while using the operating room as a classroom. The effects of "learning on the job" on patient safety, however, are a worry [4]. Additionally, legislation to restrict the working hours of surgical residents has been proposed in the USA and Europe. As a result, simulation training has gained popularity [1,4]. 

It is not surprising that more surgical problems arise in the early stages of the surgeon's learning curve. By utilizing alternate training techniques for skill acquisition, we have an ethical obligation to minimize patient harm. The role of simulation is to facilitate training in a secure and risk-free environment by removing the learning curve from the operation room. One can make mistakes, grow from them, and learn to perfection, which is reflected upon without harming a single patient [1]. Our aim is to determine the importance of simulation in the training of surgeons as well as identify areas for future research and development.

## Review

The need for surgical simulation

Due to inexperience during the early training period, surgical trainees can cause inevitable patient harm, which is preventable or terminated with the guidance and supervision of experienced senior surgeons. Quality improvement is obligatory to obtain patient safety and quality care [5]. Nevertheless, patients are not training commodities; this is why there is a need for surgical simulation training [5,6]. Innovations in surgical simulation devices are useful for training and enhancing surgical skills without creating any risk to the patient. Surgical trainees can have self-centered education and training opportunities with continuous assessment of their wide skill range and video feedback availability to improve performance and error management [5,6]. Errors are not welcomed in the operating suite since they can be fatal to patients; however, in a simulated environment, training errors are allowed so that trainees learn to progress and rectify the error. Surgical simulation can be used by senior surgeons to improve existing skills and become proficient in new procedures [5].

Definition and types of simulation training in surgery

Definition

Surgical simulation is achieved when aspects of real-life situations are convincingly replicated in the form of simulations to provide surgical training [7]. Evolving over the past several decades, simulation in surgical education has created a path for trainees to acquire new skills and knowledge [8]. By overriding the need for direct human contact, it represents a means through which surgeons can enhance their skill set without involving patients [7,9].

Types of Surgical Simulation 

There are several models and devices that use simulation as a means for surgical training [10]. Broadly, simulators in surgery can be divided into organic and inorganic [5]. Whereas organic simulators, either live or cadaveric, are high-fidelity and uphold realism, inorganic models tend to be low-fidelity and can be further subdivided into synthetic and electronic [5,11].

Organic-animal: Notwithstanding the fact that differences exist between human and animal anatomical structures, training on live animals helps improve technical skills, reduces complication rates, and provides an environment that warrants theater-like communication and teamwork between participants [5,12]. The most commonly used models include canines, porcines, and baboons [10,13,14]. In both America and Europe, animal models are frequently employed. The UK's legal system allows the use of dead animal tissue but restricts the use of live animal tissue for ethical concerns. Disadvantages include high costs, the need for pain control, infectious disease spread, perioperative monitoring, and ethical and legal concerns [15-17].

Organic-cadaver: Organic-cadaveric training provides the highest standard of surgical simulation because it provides an excellent opportunity to understand and comprehend the intricate details of human anatomy [10,18,19]. Some commonly simulated procedures include laparoscopy, endoscopy, and saphenous vein cutting [20,21]. Cadavers are most typically utilized for specific courses in both the United States and the United Kingdom. Disadvantageous aspects of this modality include acquisition and maintenance costs, limited availability, and distortion of loco-regional anatomy due to preservatives like formalin. The absence of physiological considerations encountered during surgery is also an issue [22-26].

Inorganic-synthetic: Synthetic simulators are remarkably beneficial in developing the hand dexterity and motor skills needed to dissect, suture, grasp, or clip structures [27]. As the name suggests, they are usually made of synthetic materials such as plastic, rubber, and latex [27]. They are also particularly advantageous for simulating complex surgeries like aneurysm repairs, joint replacements, fracture fixations, and laparoscopic surgery [28]. Nonetheless, they can be fairly expensive, time-consuming to manage, and do not achieve the same level of anatomical exactitude as cadavers or live animals [5].

Inorganic-electronic: Technological advancements have enabled the use of high-fidelity computer-based simulations due to their impressive tactile feedback and user-friendly interface [27,29]. Real-time haptic feedback ensures that trainees are made aware of errors in both time management and surgical technique [22]. By changing the observed territory, the VIPAR system has provided a means of long-distance surgical tele-collaboration [30]. Moreover, systems that involve patient-specific VR simulators create a surgical field using the patient’s own imaging data, which can further help reduce intraoperative surgical errors [31].

Simulators functions

Simulators are essential for achieving training goals since they have supplementary functions and instructional features [32,33]. The briefing function prepares trainees, while the demonstration function showcases ideal performances done by the instructor or by automated displays [33]. The practice function is central to training [34], setting initial conditions swiftly, offering scenario selection, and adjusting task complexity based on performance. Automated controllers or instructor-provided performance data inform operators, and the freeze feature stops an exercise component or the entire system [33].

Performance measurement and analysis provide quantifiable results for valuable trainee evaluation. Learning enhancement utilizes record and replay features for feedback, while performance alerts offer immediate coaching [33]. The system malfunction and failure functions are vital, allowing trainees to practice emergency responses and system failure management. Trainees gain essential skills by employing these simulator capabilities, better preparing them for real-world challenges in a concise and safe training environment. Instructors benefit from an array of information through the instructor station, enabling real-time monitoring and comparison among trainees within a group [35].

Theoretical framework of simulation training

Simulation training is a powerful educational method that aims to recreate real-world scenarios in a controlled environment to enhance learning and skill development. The theoretical framework of simulation training is based on principles from several disciplines, including education, psychology, and human factors. It relies on a few major learning theories, such as social-cognitive, behavioral, constructivist, cognitive load, and experiential learning [36-38]. The following are the key components of the theoretical framework of simulation training:

Experiential learning: David Kolb’s Experiential Learning Theory signifies the value of solid experiences during the process of learning. Simulation training provides apprentices with immersive experiences where they can practice skills and face the consequences of their actions within a safe environment. The experiential learning cycle has four steps: concrete experience, observations and reflections, the formation of abstract concepts and generalizations, and testing the implications of concepts in new situations. Learners may better internalize the knowledge and improve their performance by following this cycle [38].

Cognitive load theory: John Sweller’s cognitive load theory emphasizes how learners process information and manage the mental load during learning. In simulation training, careful consideration is given to the complexity of the scenarios presented to learners. By managing cognitive load through well-designed simulations, trainers can ensure that learners can effectively process and retain essential information [39].

Constructivism: It is an important learning theory that suggests that learners can actively build their own perspective by integrating new insight with their existing mental models. Through the process of actions, reflections, and constructions, knowledge is achieved. In simulation training, learners engage in hands-on experiences and reflective learning, allowing them to build upon their prior knowledge and experiences. Simulations encourage learners to figure out problems and solve them in a realistic context, enhancing their understanding and retention of concepts [40].

By drawing upon these theoretical frameworks, simulation training can create effective learning experiences that engage learners, promote active participation, and lead to better skill development and performance in real-world contexts. It offers a valuable approach for training professionals across various domains, including healthcare, aviation, the military, emergency services, and more.

Theoretical underpinnings supporting the effectiveness of simulation training

Health care simulation refers to an expensive and valuable educational method that enables healthcare professionals to acquire knowledge, refine their skills, and build expertise in a secure and well-organized setting, all while safeguarding from any potential risks.

However, simulation-based training can become ineffective and reduced to “just an experience” without acquiring knowledge or skill refinement [41]. To delve deeper into this topic, we can divide it into three components: 

Individual: Each person has their own mental model, which represents their mental representation of how the world works. This mental model is composed of interconnected memories and knowledge that create an expectation about the future [42]. Simulation training has the capacity to inspire students, improving their hands-on skills. Subsequently, it motivates them to engage in the learning process actively [43]. Simulation necessitates that every participant apply their existing mental frameworks during the situation [11]. These mental models influence how people interpret and respond to different situations. The simulation provides a great opportunity for an individual's ability to recognize specific areas where they desire growth [44].

Experiences: This is based on a training that might be existent and present or could also be empiric; however, the real process of learning is not going to be solid during the training and will be subsequent in the subsequent discussion and analysis [11]. Debriefing is a discussion that happens right after the experiences and is where the teachers can study the individual and group abilities and difficulties [45]. Each session of training or class in simulation enables the determination of knowledge gaps but also improves the skills of students in every session [46].

Environment: For effective learning, the environment should have mentors with critical skills and tools to achieve educational feedback that can highlight all the positive aspects of the experience to motivate the student, but at the same time, the negative ones will become a possibility to improve them. As a result, feedback encourages students to reflect on their strengths and weaknesses, enabling them to focus on improvement [41].

In conclusion, healthcare simulation is a strong educational instrument, and the main goal of this is to give the students an opportunity to apply what they have learned through theory. Before taking it to the patients, they will already have certain tools they acquired during the practice [11]. This makes it easier for students to identify the knowledge gaps that they must fill to improve patient outcomes [41]. Moreover, simulation brings a wonderful opportunity for students to acquire skills, gain confidence, and experience success before working with real patients, especially when their clinical exposure is limited [47].

Benefits of simulation training in surgical education

Trainee doctors will gain technical expertise as soon as they treat patients. Patients should not be subjected to the risk of damage when alternative training approaches are available for skill improvement [48]. The use of simulation to teach technical skills to surgical trainees has become more prevalent as a result of residents working fewer hours, training programs being shorter, operating rooms being used less frequently, and ethical obligations to prevent patients from injury [49-51]. 

The resident's and the attendees' confidence in the trainee's competence to operate is increased by early exposure to fundamental surgical methods [52]. Simulation makes sure that some training has taken place and enhances decision-making before learners treat actual patients [9]. Through simulation, trainees can acquire and practice the skills necessary to incorporate new surgical technologies and innovation into their surgical repertoire, as well as the effects of errors [53]. Simulation also allows for error.

Teaching in the operating room also takes a lot of time and resources. According to research by Bridges et al., resident education-related increases in operational time would cost 53 million dollars annually [54]. The Minimally Invasive Surgical Trainer-Virtual Reality (MIST-VR) system is made up of a low-fidelity system designed to teach general laparoscopic skills, a system that teaches component procedural skills, and other systems that replicate all the procedures and allow training to be done at various levels without the need for an expert [55,56].

According to an article in the American Journal of Surgery, the price to set up a simulation lab ranges from $100,000 to several million dollars. The authors also assert that an annual average of $12,000 to $300,000 might be used to pay for supplies and running expenses. As a result, pharmaceutical corporations routinely provide funding for training programs for healthcare personnel. However, the majority of experts agree that investment funds will matter [57], regardless of how the simulation labs are supported.

Assessment and validity of simulation training

There are numerous evaluation measures that can be used to evaluate the learning curve for knowledge and thought abilities, and there has been increasing interest in evaluating the validity of these assessment instruments [58].

Methods of Evaluating Simulation-Based Surgical Training

**Figure 1:**
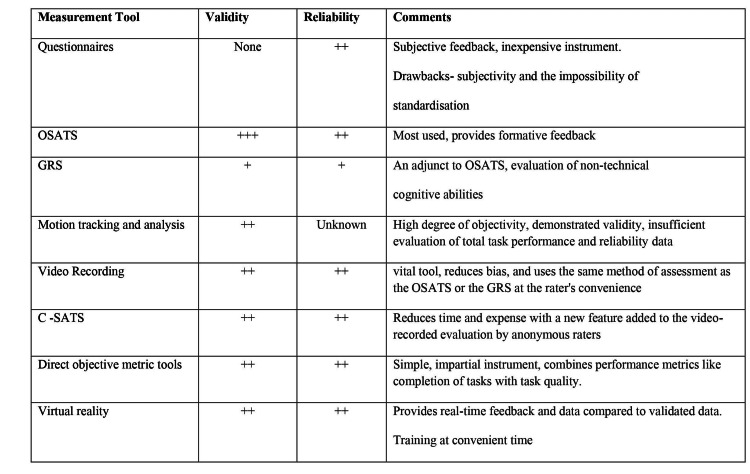
Methods of evaluating simulation-based surgical training

With the emergence of simulation-based surgical training, it is essential to have a variety of evaluation techniques that concentrate on helping trainees attain a specific level of performance and gain a set of abilities that are necessary to conduct certain procedures [58]. The quality of surgical education must be increased, as must professionalism and competence. The following requirements should be met by the measurement tools used in the assessment: they must be usable, practical, objective, valid, and reliable to be acknowledged as standards [5,58]. Brief descriptions of the methods of simulation assessment are mentioned in Figure 1. 

Questionnaires and surveys: Surveys are created so that instructors can get trainees' opinions about how they personally feel about or how much they know about a certain medical technique. Even though they are relatively inexpensive instruments, questionnaires have several intrinsic drawbacks, including subjectivity and the impossibility of standardization [5]. 

Objective structured assessment of technical skills (OSATS): The first grading instrument that evaluated the performance of a quantifiable surgical skill or task in a surgical simulator was the Objective Structured Assessment of Technical Skills (OSATS). A checklist of surgical movements that have been identified as crucial components of the operation is used by impartial observers to assess the trainee's performance, and formative feedback can be offered, which can considerably improve training [5,59].

Global rating scale: The Global Rating Scale (GRS) is a frequently used technique for evaluating surgical abilities. It measures the surgical finesse that is typical of surgeons when they conduct certain procedures. This assessment is incorporated into OSATS by some researchers. GRS offers a thorough evaluation that evaluates non-technical cognitive abilities using both objective and subjective criteria [5].

Motion tracking and analysis: Motion tracking and analysis appear to be a neutral and dependable method for testing operational capabilities in terms of accuracy and efficiency of motion during surgical procedures. As sensors, motion tracking devices can be worn on the hands or attached to surgical instruments. 3D coordinates are used to record the motions to track various motions [59].

Video recording: Videotaping can be used to assess a trainee's competence. Video-based evaluation is a valuable tool for evaluating surgical performance since it uses the same assessment procedures as the OSATS or the GRS at the rater's convenience. Bias can be reduced by having numerous raters watch and evaluate the same video footage [5].

Crowd-sourced assessment of technical skills (C-SATS): C-SATS is a new addition to the video-recorded assessment of clinical abilities. In this approach, video-recorded surgical techniques can be evaluated by decentralized, anonymous, and independent online crowds of raters; some of these observers might not have medical knowledge [5].

Direct objective metric measures: Simply and impartially assessing a tangible component of a skill using a widespread metric system in research on a large scale or high-stakes examination has promise for enhancing accuracy, dependability, therapeutic significance, and usefulness while reducing time and expense. For example, the mechanical durability of a knot or the fixation of a fracture [5]. 

Virtual reality: The capacity to produce results is an appealing aspect of our minimally invasive surgical instructor augmented reality system. It delivers real-time feedback and compares the results to validated data. VR instructors may let trainers train on their own in their downtime, and this practice can subsequently be included in the organized syllabus [59]. 

Reliability and validity of simulation-based assessment tools

The learner should receive valid and reliable findings from the numerous assessment instruments used to evaluate simulation-based surgical training. A novel assessment should compare its findings to an existing ideal that is used as a benchmark for others. However, this is not always the case, so comparisons should be made with other assessment instruments that have a similar purpose [59].

The consistency of a measurement is related to reliability [60]. For instance, a learner completing a test to gauge the efficacy of chest compressions ought to come up with findings that are roughly consistent each time. 

**Figure 2:**
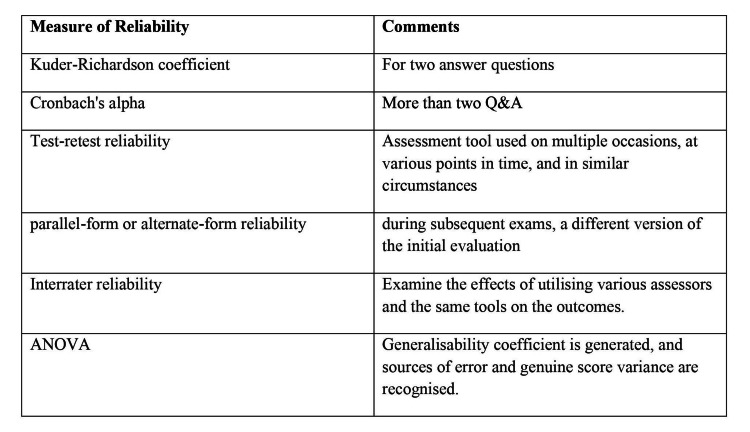
Measure of Reliability

For two questions or more and for fewer, the Kuder-Richardson coefficient and Cronbach's alpha approaches are employed to assess reliability. Internal consistency tests are conducted using these. Test-retest reliability is determined by a connection between the measurements recorded at different times when learners are given an assessment tool more than once, at varied times, and in comparable situations. Test-retest reliability is identical to parallel-form or alternate-form reliability, with the exception that learners are given a different version of the initial assessment during future assessments [61]. Interrater reliability is a different type of measurement that looks at how different assessors using the same tools affect results [61]. ANOVA (Analysis of Variance) testing is another technique for calculating a generalizability coefficient. The amount of measurement error owing to each potential element, such as variances in question wording, learner attributes, raters, or the period between assessments, is determined using this method [60]. Measures of reliability are discussed, in short, in Figure 2. 

An evaluation tool's validity is determined by how well it captures the goals for which it was designed. Evidence supporting the use of the evaluation tool in a specific setting is necessary to establish validity. It is not always essential to build new tools, but they must be appropriate for program activities and report reliability and validity [60]. 

The five sources of evidence for validity are content validity, response process, internal structure, relationship with other variables, and consequences [59,60].

**Figure 3:**
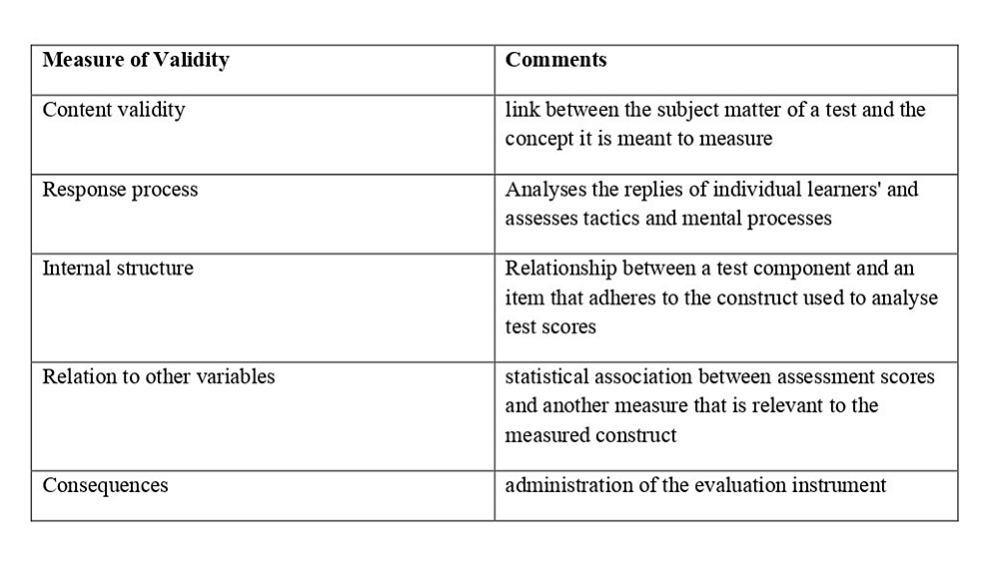
Measure of Validity

A test's content validity is defined as the "relationship between a test's content and the construct the test is intended to measure". This refers to the concepts, phrasing, and format of the items in an assessment tool. If students get better after getting more training, the validity of the evaluation technique is supported [60]. The response process is the analysis of the replies to the assessment, methods, and ideas used by students. It may be possible to identify sources of inconsistency by examining the variation in response patterns among various learner types [60, 61]. The internal structure of the assessment tool is the links between test items and test components, on which the recommended test score interpretations are based [61]. Relation to other variables - when two measures have a high positive correlation, they are measuring the same construct; nevertheless, when they have a weak link, they should be independent [60,61]. Consequences are the results of utilizing the evaluation instrument, which might be favorable or negative, intentional or unforeseen. This could either support or disprove the validity of score interpretations [60]. The validity measures described above are briefly shown in Figure 3.

Challenges and potential biases in assessment tools

The numerous assessment instruments have been covered, along with methods for evaluating their reliability and validity. It is crucial to think about how to include simulation in training. "New technology must continually seek to improve the quality of teaching and learning and not simply to open access to new information," said Satava, a pioneer in the field of virtual reality [62].

According to Kneebone, simulation-based training should be a proactive procedure. Understanding the new technology, creating a curriculum, and finally integrating it into surgical training are thus the main challenges [63]. Some potential barriers to validity include low dependability, misalignment of the assessment with learning objectives, and interface issues such as poor instructions or a lack of computer expertise for computerized examinations. These should be addressed and removed to obtain valid evidence [61]. By designing an evaluation process that provides no room for assessor interpretation, such as by adopting a consistent rubric, subjectivity should be eliminated. However, even using this strategy, personal bias would still exist.

Various studies supporting simulation training

Published literature demonstrates that simulation has been accepted as a reliable approach to evaluation. In comparison to traditional clinical education, competence training in virtual reality, for example, revealed significant gains in operative ability during actual surgery. Virtual reality training, according to Larsen et al., shortened the learning curve during laparoscopic salpingectomy [64]. The simulator can help you avoid the early stages of the learning curve. The operation was shortened in half, and the skills obtained in virtual training could be implemented in real-world operations. The results of the study suggest that proficiency-based training should be included in a comprehensive surgical training program before trainees undergo actual surgery on actual patients, improving both the efficiency of the operating room and patient safety [64].

Grantcharov et al. discovered that training in a virtual environment improved technical skills. Laparoscopic patient training has shown significant difficulties that have an impact on patient safety [65]. Previous research found that people who underwent virtual reality training had much better psychomotor abilities than those who did not [64,65].

Cragg et al.'s study reveals that surgical cognitive simulation (SCS) could be a beneficial adjunct to technical simulation. SCS is designed to help surgeons focus on their sensory capabilities and practice the procedure before conducting it. Theories from the fields of psychology and neuroscience are the foundation of SCS. By employing the "simulation center in the brain" to mentally practice the technique, surgeons may be able to shorten their learning curve and spend less time in the skills lab. After receiving introductory training from a facilitator, trainees are free to use it whenever and wherever they like [66].

In addition to retrospective studies that have shown how simulation-based training for cataract surgery resulted in a 38.1% reduction in posterior capsule rupture (PCR) rates for UK trainees [67], the randomized OLIMPICS trial further demonstrated that the surgical competence of trainees also underwent a near three-fold increase [68]. The benefits of the intensive five-day course in small incision cataract surgery (SICS) were noted to have endured for over a year with markedly reduced complications rates [69]. In a different RCT trial, surgical cognitive simulation (SCS) training significantly improved trainee surgical performance both subjectively and objectively [66], mirroring the findings of another RCT in which the virtual reality simulator (VRS) trained group consistently ranked at the top in several assessments [69].

In the study by Dean WH et al., second, third, and fourth-year trainees underwent a surgical simulation training program, a parallel-group efficacy trial, for almost 15 months. Eventually, in the training program, the trainees' competency and surgical skill confidence were remarkably increased as surgeons. The intense simulation training provides a safe and calm setting to practice complex and meticulous surgical skills without causing any harm to patients [70]. Another study by Annoh R et al. observed minimal incidence and decreased operating time in surgical residents who underwent simulation-based surgical education (SBSE). Repetitive surgical simulation training can improve speed and fluidity in general surgical skills in comparison to conventional training [71]. Nilsson C. et al. compare learners who received structured simulation training to a group of trainees who did not receive any simulation training in a single-center randomized control research. Significantly faster and less time-consuming skill proficiency was noticeable in simulated trainees. Despite being anxious in the operating room (OR) for the first time, simulated trainees completed the surgery on time, demonstrating the effectiveness of surgical simulation training [72].

Comparison with traditional training methods

Contrasting Simulation Training With Traditional Methods

Traditional surgical training involves senior surgeon supervision in the operating room along with printed and audio-visual documents [73]. Discussion and performance alignment between the senior surgeon and surgical trainee are two of the key components of effective training [74]. In simulation-based training, the trainees will have full control over a variety of clinical scenarios and settings to take risks and make errors without causing harm to live patients by going further into the procedure [6]. 

Strengths and Weaknesses of Each Approach

Formative assessment and feedback from the senior surgeon are informative for the trainee in the traditional training setting and help rectify their shortcomings by improving them [74]. However, due to the drawbacks of high training costs and ethical issues, traditional surgical training is being rejected around the world gradually [73]. Performance misalignment and failure of responsiveness by the novice trainee will lead to an increase in operating time and an increase in the risk of morbidity [73,74]. Simulation training provides a safer and more efficient instrument handling technique, and it is evident that it brings out a minimal chance of error. In contrast to traditional apprenticeship training, simulation training skills may not be applicable due to anatomical variations that trainees might encounter in real-life surgery [75].

Simulator training advantages 

Some advantages of simulators are highlighted, as they benefit trainees, instructors, and the overall medical system [35]. Simulators allow practice tasks under conditions resembling real-life scenarios and infrequent but critical situations, such as system failures or accidents that could cause significant social and environmental damage. Simulators can contribute to economic savings for the system [35]. 

Furthermore, simulators increase coverage, accommodating a greater number of trainees and providing opportunities for multiple practice attempts or extended training periods [35]. Instructors can assess performance through quantitative measurements, enabling the development of highly effective training strategies customized to meet the unique requirements of each trainee [35]. This equates to individualized training, where the simulator can adapt to the specific needs of each trainee and adjust the difficulty as the trainee improves [35].

Ultimately, simulation training fosters students' self-assurance in handling comparable situations [76], enhancing their competence and preparing them to manage real-life scenarios in the future effectively [77].

Challenges and future directions 

In the past three decades, simulation has developed into a crucial tool for maintaining patient safety while training medical professionals. An immersive, realistic method of acquiring technical skills is through simulation [78]. There are a few negative aspects to it, though. A few of the disadvantages of immersive visual reality systems include the visual quality, level of presence, cyber-sickness, haptic realism, device-related issues (such as battery life and wireless technologies), and cost considerations [79,80].

Recent personal experience with a laparoscopic simulator has shown that when errors are made, authenticity can degrade suddenly and quickly. For instance, bleeding may occasionally show up not only visibly on the screen but also by shaking the trocars erratically. Without haptics, training on virtual simulators can cause one's pulling and pushing forces, which are frequently greater than what the tissue needs, to be distorted [80]. A good method of simulation training is using virtual reality simulators with haptics and simulated patients. The availability of these facilities is limited, though, and a typical session might include an exercise involving stacking sugar cubes and box trainers [78]. The degree of expertise or competency is one area that needs clarification as medical education transitions to a competency-based paradigm.

## Conclusions

Simulation training has disrupted surgical education, replacing outdated methods with risk-free, skill-enhancing practice. Shifting from the "look one, do one, teach one" approach, simulation offers a secure environment to learn and execute surgical procedures. Various models, from animals and cadavers to electronic versions, foster experiential learning and align with established theories. Trainees gain technical expertise, decision-making skills, and self-confidence, identifying strengths and areas for growth. Studies affirm simulation's positive impact on participants, minimizing complications and elevating surgical proficiency.

Challenges include assessing the validity and reliability of tools, managing bias, and adapting to technological progress. Future trends envision advanced virtual reality systems for personalized, immersive learning and competency-based training that prioritize skill management and patient safety. Simulation training reflects medical advancement, prioritizing patient well-being and healthcare quality. The surgical community recognizes its potential, cultivating skilled and patient-focused surgeons for optimal outcomes. This innovative education model's transition to real-world practice underscores its value for healthcare professionals and patients, ultimately shaping a safer, more proficient future.
